# Untargeted metabolomics reveals a mild impact of remote ischemic conditioning on the plasma metabolome and α-hydroxybutyrate as a possible cardioprotective factor and biomarker of tissue ischemia

**DOI:** 10.1007/s11306-017-1202-2

**Published:** 2017-04-17

**Authors:** Mia Roest Laursen, Jakob Hansen, Casper Elkjær, Ninna Stavnager, Camilla Bak Nielsen, Kasper Pryds, Jacob Johnsen, Jan Møller Nielsen, Hans Erik Bøtker, Mogens Johannsen

**Affiliations:** 1 Department of Forensic Medicine, Section for Forensic Chemistry, Aarhus University, Aarhus N, Denmark; 2 Department of Cardiology, Aarhus University Hospital, Aarhus N, Denmark

**Keywords:** Remote ischemic conditioning, Ischemic preconditioning, Untargeted metabolomics, α-Hydroxybutyrate, Cardioprotection, Ischemia biomarker

## Abstract

**Introduction:**

Remote ischemic conditioning (RIC) is a maneuver by which short non-lethal ischemic events are applied on distant organs or limbs to reduce ischemia and reperfusion injuries caused by e.g. myocardial infarct. Although intensively investigated, the specific mechanism of this protective phenomenon remains incompletely understood and in particular, knowledge on the role of small metabolites is scarce.

**Objectives:**

In this study, we aimed to study perturbations in the plasma metabolome following RIC and gain insight into metabolic changes by the intervention as well as to identify potential novel cardio-protective metabolites.

**Methods:**

Blood plasma samples from ten healthy males were collected prior to and after RIC and tested for bioactivity in a HL-1 based cellular model of ischemia–reperfusion damage. Following this, the plasma was analyzed using untargeted LC-qTOF-MS and regulated metabolites were identified using univariate and multivariate statistical analysis. Results were finally verified in a second plasma study from the same group of volunteers and by testing a metabolite ester in the HL-1 cell model.

**Results:**

The analysis revealed a moderate impact on the plasma metabolome following RIC. One metabolite, α-hydroxybutyrate (AHB) however, stood out as highly significantly upregulated after RIC. AHB might be a novel and more sensitive plasma-biomarker of transient tissue ischemia than lactate. Importantly, it was also found that a cell permeable AHB precursor protects cardiomyocytes from ischemia–reperfusion damage.

**Conclusion:**

Untargeted metabolomics analysis of plasma following RIC has led to insight into metabolism during RIC and revealed a possible novel metabolite of relevance to ischemic-reperfusion damage.

**Electronic supplementary material:**

The online version of this article (doi:10.1007/s11306-017-1202-2) contains supplementary material, which is available to authorized users.

## Introduction

Ischemic conditioning is a maneuver by which a series of short intervals of ischemia and reperfusion is utilized to reduce tissue damage following a prolonged ischemic event as e.g. a myocardial infarct. It protects tissue against ischemia and reperfusion injuries not only locally but also remotely from the organ being conditioned (Heusch et al. 2015). In the clinical setting, remote ischemic conditioning (RIC) is induced by applying brief cycles of ischemia and reperfusion before, during, or immediately after reperfusion of the ischemic organ. Remote conditioning is performed by a simple blood pressure cuff on outer limbs that is inflated and deflated in e.g. four short intervals of 5 min. The procedure has been shown to improve myocardial salvage and clinical outcome (Bøtker et al. 2010; Sloth et al. 2014).

A first phase of protection follows immediately after the induced stimulus and lasts 2–3 h. After 24 h a second phase of protections appears which persists up to 2–3 days after conditioning (Hausenloy and Yellon 2009). While the second phase of protection involves transcriptional changes, a neural component as well as a humeral component seems involved in the first phase of protection (Breivik et al. 2010; Jensen et al. 2012; Lim et al. 2010; Shimizu et al. 2009). Involvement of circulating humeral factors is supported by the fact that the protective effect is transferable between individuals via blood or coronary effluent and even between species (Dickson et al. 1999; Huffman et al. 2008; Shimizu et al. 2009; Skyschally et al. 2015). Owing to the rapid onset of the first phase of protection, it seems reasonable to assume that the protection is mediated by regulation of intracellular processes, and that the circulating mediators are likely latently present in the biological system before conditioning i.e. the process does not involve transcriptional activity (Pérez-pinzón 2004). Different studies have estimated the circulating factor(s) as being of a size of 3.5–30 kDa, temperature labile, and hydrophobic (Breivik et al. 2010; Serejo et al. 2007; Shimizu et al. 2009). Furthermore, protease inhibitors were shown to stabilize the biological activity of effluent from rat hearts, indicating that small proteins or peptides could mediate the circulatory effects (Serejo et al. 2007). Similarly, a range of studies has addressed how intracellular signaling pathways are activated during ischemic conditioning, identifying e.g. protein kinase C (PKC), phosphatidylinositol (4,5)-bisphosphate 3-kinase (PI3K), and signal transducer and activator of transcription (STAT) as central components (Heusch 2015; Mayr et al. 2009).

While much attention has been paid to these high-molecular weight compounds and their possible role as circulating factors involved in RIC, only little is known about the influence of ischemic conditioning on the human plasma metabolome. Being downstream of the genome and the proteome, the metabolome directly reflects a given phenotype (Mayr et al. 2009; Yin and Xu 2014). Within recent years metabolomics has successfully been applied to get detailed insight into metabolic changes following e.g. disease (Madji Hounoum et al. 2016; Yin and Xu 2014). Animal studies of tissues or plasma (rat and mouse) have suggested ischemic conditioning to regulate metabolites involved in glycolysis, glutathione oxidation balance, synthesis of glycogen and amino acids, as well as fatty-acids and shingolipid metabolism (Kouassi Nzoughet et al. 2017; Nadtochiy et al. 2015; Zhou et al. 2015). At this point, a full understanding of the metabolic changes and how they are involved in local as well as remote protection does, however, not exist.

In the present study, we used an untargeted metabolomics approach to study changes in the plasma metabolome of humans during the initial phase of protection following a RIC intervention. This was expected to improve the understanding of the biology involved in the protection as well as to identify potential novel cardioprotective metabolites.

## Materials and methods

### Materials

LC-MS grade methanol and acetonitrile as well as adenosine (CAS# 58-61-7), hypoxanthine (CAS# 68-94-0), succinyladenosine (CAS# 4542-23-8), pantothenate (CAS# 79-83-4), propionylcarnitine (CAS# 17298-37-2), sphinganine 1-phosphate (CAS# 19794-97-9), sphingosine 1-phosphate (CAS# 26993-30-6), uridine monophosphate (UMP, CAS# 58-97-9), α-hydroxybutyrate (AHB, CAS# 600-15-7), β-hydroxybutyrate (BHB, CAS# 300-85-6), 2-hydroxy-3-methylbutyrate (CAS# 4026-18-0), decanoylcarnitine (CAS# 1492-27-9), dodecanoylcarnitine (CAS# 25518-54-1), claycomb medium, fetal bovine serum (FBS), penicillin–streptomycin, norepinephrine, ascorbic acid, glutamine, trypsin-EDTA, trypsin inhibitor type I-S, soybean, Dulbecco’s PBS, gelatin, fibronectin, glucose and all salts used in buffer solutions for cell experiments were from Sigma-Aldrich. ^13^C_4_ 3-Hydroxybutyric acid was purchased by Cambridge Isotope Laboratories (Andover, MA, USA), (R)/(S)-methyl-2-hydroxybutyric acid (MHB) from Small Molecules, inc. (Hoboken, MA, USA), and propidium iodide and hoechst33342 from Thermo Fischer Scientific Inc. (Waltham, MA, USA). MilliQ water was produced freshly using a Direct-Q-3 apparatus (Millipore, Bedford, MA, USA). Finally, HL-1 cardiomyocytes were a kind gift from Dr. Claycomb (Louisiana State University, New Orleans, LA, USA).

### Human study and sample collection

**Fig. 1:**
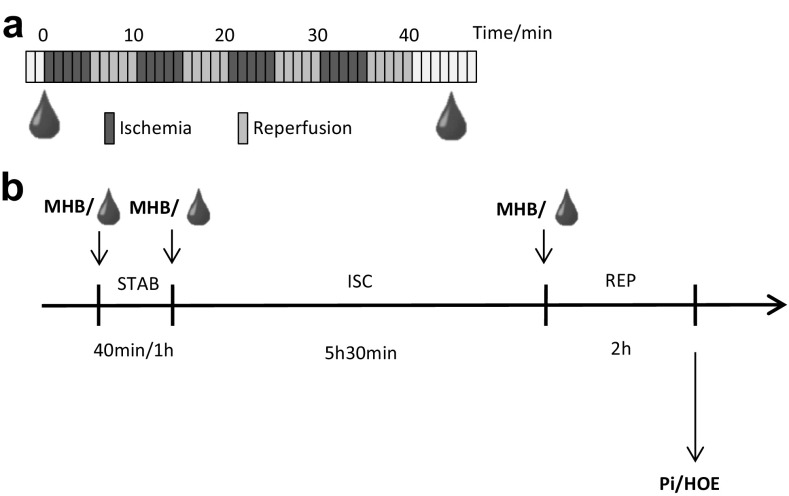
Study set-up showing the sample collection strategy (**a**) in which blood samples were collected before and just after 4 cycles of 5 min ischemia and 5 min reperfusion and study set-up for cell studies (**b**) adding either plasma or (R)/(S)-methyl-2-hydroxybutanoate (MHB) to buffer solutions during 40 min or 1 h stabilization (STAB), 5 h 30 min ischemia (ISC) and 2 h reperfusion (REP). Cellular damage was measured by propidium iodide/hoechst33342 (PI/HOE) staining

Two separate sets of blood samples were obtained from the same volunteers constituting a calibration set for data inspection and a validation set in which findings were confirmed. The two sets were collected, using an identical protocol, at least 1 month apart from ten healthy, lean men in the age of 18–45 years. Inclusion criteria required that no physical activity was performed 48 h prior to sampling and that intake of drugs, alcohol, and caffeine was avoided 24 h before this time. All samples were collected after minimum 12 h of fasting. RIC was induced by ischemia/reperfusion of the upper arm using the AutoRIC™ device (CellAegis Devices Inc., Toronto, ON, Canada). The RIC maneuver consisted of a total of four consecutive cycles of 5 min inflation of a blood pressure cuff to above arterial systolic pressure and 5 min deflation. Blood samples (9 mL) were collected in lithium heparin tubes from the contralateral arm immediately before and after the RIC maneuver (Fig. 1a). Immediately after sample collection, plasma was recovered by centrifugation (500 g, 5 min, 4 °C) and kept on ice until the end of sample collection. Samples were stored at −80 °C until analysis.

Following the same procedure, four blood samples from random participants were also drawn in tubes containing clotting activating agents and left to coagulate for 20 min before centrifugation (500 g, 5 min, 4 °C) to recover serum. Serum samples were stored at −80 °C until use.

### Plasma sample preparation for untargeted metabolomics

For the calibration set, plasma samples were thawed and aliquots transferred to a clean 96-well plate in duplicates (160 μL). Ice-cold methanol (600 μL) was added to each well and the plate was shaken (1000 rpm, 5 min, rt) and incubated (10 min, 4 °C). The supernatant was separated by centrifugation (2000*g*, 10 min, 4 °C) and transferred to glass inserts in a separate 96-well plate. Ice-cold methanol/water (80:20) (600 μL) was added to the pellet and the extraction was repeated. Supernatants were pooled and evaporated to dryness under N_2_. Samples were re-suspended in Millipore water, 0.1% formic acid (160 μL), and a 10 μL aliquot of each sample was combined to obtain quality control (QC) samples, before the plate was sealed for analysis. Two samples were excluded due to errors in sample preparation giving a total of 38 samples. Samples included in the validation set were prepared in a similar manner, however only one aliquot was transferred to the 96-well plate, resulting in 20 samples.

### Equipment used for untargeted metabolomics

For untargeted metabolomics, reverse phase (RP) separation was performed by an Acquity UPLC I-Class system from Waters Corporation (Milford, MA, USA) while detection used MaXis Impact qTOF-MS instrumentation from Bruker Daltonics (Bremen, Germany). The qTOF-MS instrument was operated in positive electrospray ionization mode (ESI+) using a capillary voltage of 4.0 kV and in negative electrospray ionization mode (ESI−) with a capillary voltage of 2.5 kV. The nebulizing gas pressure was 4 bar, and the drying gas flow and temperature were 11 L/min and 220 °C. HyStar, (Bruker Daltonics, Bremen, Germany) was used as a common platform to control both systems.

### Analysis for untargeted metabolomics

For untargeted analyses, separation was performed using a HSS C18 column (2.1 × 100 mm, 1.8 μm, Waters Corporation) at 50 °C. The mobile phase consisted of Millipore water, 0.1% formic acid (A1) and acetonitrile, 0.1% formic acid (B1), and a constant flow rate of 0.6 mL/min spanned the total runtime of 15 min. A gradient elution was isocratic at 0.0–2.0 min at 0% B1, linear from 0 to 40% B1 at 2.0–6.0 min, linear from 40 to 60% B1 at 6.0–6.5 min, linear from 60 to 88% B1 at 6.5–11.0 min, linear from 88 to 100% B1 at 11.0–11.5 min, linear from 100 to 0% B1 at 11.5–12.5 min and isocratic at 0% B1 for column equilibration at 12.5–15.0 min. Data were acquired from 0.2 to 11.5 min. Samples were kept at a constant temperature of 7 °C and the injection volume was 2 μL in ESI+ mode and 10 μL in ESI− mode. Scans were acquired at a rate of 4 Hz using a mass range of 50–1000 m/z. Calibration of the instrument was performed using sodium formate/acetate for positive ionization mode and sodium formate for negative ionization mode and was followed by a subsequent recalibration of all samples in DataAnalysis (Bruker) to improve mass accuracy further. Instrument control samples, containing butanoylcarnitine, palmitoylcarnitine, citric acid, glutamic acid, valine, inosine, and xanthine, were analyzed before and after analysis of samples to validate the performance of the instrument in positive ionization mode. For negative ionization mode, instrument control (IC) samples contained adenosine, citric acid, glutamic acid, hippuric acid, histidine, inosine, malic acid, succinic acid, tryptophan, and xanthine. QC samples were analyzed before, after, and in between each ten samples to check instrument stability. A total runtime of 20 h covered all 38 randomized samples included in the calibration set as well as IC samples and QC samples. As the validation set included only 20 samples a shorter runtime of 13 h was sufficient.

### Preprocessing of data

Calibrated data were examined in DataAnalysis (Bruker Daltonics, Bremen, Germany) and converted to the file mzXML format by CompassXport (Bruker Daltonics, Bremen, Germany). Features, described by an m/z value, a retention time, and an area under curve, were extracted with XCMS (Smith et al. 2006) using R project, version 3.2.0 (http://www.R-project.org). Removal of calibration scans (30 scans) was performed prior to peak detection. CAMERA (Kuhl et al. 2012) was used for grouping of features and annotations of isotopes and adducts. Peak detection was performed using the CentWave algoritm (Tautenhahn et al. 2008) with 12 ppm resolution and with a signal-to-noise threshold of six. Retention time correction was performed with the Obiwarp algoritm (Prince and Marcotte 2006). Selected peaks should be present at least in 50% of the samples of either group (before or after the RIC intervention) to be included for further analyses.

### Validation of analysis and instrument performance

Representative chromatograms can be seen in Fig. S1. Instrument stability was confirmed by clustering of the QC samples in a principal component analysis (PCA) score plots (Fig. S2). Furthermore, a selected number of metabolites were required to meet the following predefined criteria [mass-to-charge deviation (m/z) <10 ppm, retention time deviation <5 sek, coefficient of variance in QC samples (CV(QC)) < 30%] in both instrument control samples and QC samples (Tables S1, S2).

### Selection and identification of features

In accordance with reported guidelines, features in the calibration set with CV(QC) > 30% were excluded in the further data handling (Gika et al. 2014), while the remaining data were transferred to SIMCA 13.0 software (Umetrics, Sweden) for multivariate data analysis. Based on variable influence on projection (VIP) scores and coefficients, an orthogonal partial least squares discriminant analysis (OPLS-DA) model was optimized by removal of features irrelevant for the separation between plasma collected before and plasma collected after the RIC stimulus. The optimized model provided VIP scores, and features were selected for identification when satisfying demands of having area under curve >5000 (XCMS, mean of duplicates) and 0.9 > fold change > 1.1 (pairwise comparison) as well as either p < 0.05 (paired t test) to indicate a significant change or VIP > 1 (SIMCA) suggesting an importance in the multivariate data analysis. The paired t test used the average of the replicate analysis results in the calibration set and the single analysis results in the validation study. Deduction of possible precursors was guided by grouping and annotations made by XCMS and CAMERA. In order to ensure the relevance of the selected features in regard to the RIC stimulus, features showing a similarly directed regulation in both calibration and validation set were considered valid, while inconsistencies discarded the features from further identification. For the validation set VIP scores were deduced from an OPLS-DA model based on all features detected with CV(QC) < 30%.

For tentative identification, selected features were searched against the freely accessible databases METLIN (https://metlin.scripps.edu) and The Human Metabolome Database, HMDB (http://www.hmdb.ca/) based on their m/z value. For some features no hits were found, and a possible identification was deduced based on fragmentation patterns. When possible, final identificatio n of all features was established by comparison of m/z value, retention time, and fragmentation patterns to reference compounds. The level of identification was designated according to the guidelines of the Metabolomics Standard Initiative (Sumner et al. 2007) and is written in Table 1.

**Table 1:** Selected features

Metabolite	Ion mode	ID level	Calibration set						Validation set			Regulation	
			m/z	rt	CV(QC)	Fold	VIP	p	Fold	VIP	p^c^		
α-Hydroxybutyrate	Neg	1	103.0403	94	4	1.23	1.41	0.00001	1.35	2.67	0.00468	↑	
2-Hydroxy-3-methylbutyrate	Neg	1	117.0557	206	3	1.10	0.82	0.03013	1.01	0.26	0.49282	↑	
Acylcarnitine	Pos	3	356.2796	415	8	0.88	n.a.^b^	0.04638	0.95	0.51	0.31398	↓	
Adenosine	Pos	1	268.1045	132	3	0.69	1.24	0.05063	0.83	1.76	0.01896	↓	
Adenosine	Neg	1	266.0891	129	3	0.69	1.13	0.06785	0.82	2.16	0.02949	↓	
Decenoylcarnitine (C10:1)^e^	Pos	2	314.2326	382	6	0.78	0.89	0.01355	0.75	2.12	0.02433	↓	
Decenoylcarnitine (C10:1)^e^	Pos	2	314.2327	390	11	0.82	0.96	0.03235	0.78	2.21	0.01782	↓	
Dodecenoylcarnitine (C12:1)	Pos	2	342.2640	408	20	0.80	1.01	0.02076	0.80	1.92	0.02481	↓	
Decanoylcarnitine (C10)	Pos	1	316.2490	398	4	0.80	1.00	0.04788	0.80	1.78	0.03529	↓	
Dimethylnonanoylcarnitine (C11)^d^	Pos	2	330.2640	404	12	0.80	0.96	0.02821	0.91	1.42	0.11149	↓	
Dodecanoylcarnitine (C12)	Pos	1	344.2800	416	4	0.76	0.98	0.03012	0.83	1.46	0.04310	↓	
Hypoxanthine	Pos	1	137.0458	48	4	0.80	1.01	0.10417	0.80	1.93	0.03704	↓	
Dipeptide (Leu/Ile)^f^	Pos	2	245.1859	246	8	0.77	1.45	0.10772	0.92	0.93	0.26577	↓	
Pantothenate	Pos	1	220.1177	201	7	1.09^a^	n.a.^b^	0.02044	1.06	0.53	0.33357	↑	
Propionylcarnitine (C3)	Pos	1	218.1387	121	4	0.93^a^	n.a.^b^	0.04318	0.92	0.60	0.05926	↓	
Sphinganine 1-phosphate	Pos	1	382.2721	450	4	0.80	1.10	0.22922	0.90	1.45	0.21910	↓	
Sphingosine 1-phosphate	Pos	1	380.2567	442	2	0.87	1.06	0.28048	0.92	1.15	0.22508	↓	
Succinyladenosine	Neg	1	382.0999	201	11	1.13	n.a.^b^	0.02228	1.03	0.24	0.66725	↑	
Tetradecadienoylcarnitine (C14:2)^e^	Pos	2	368.2797	414	8	0.78	0.87	0.03150	0.75	1.85	0.01766	↓	
Tetradecenoylcarnitine (C14:1)^e^	Pos	2	370.2957	424	4	0.80	1.02	0.03436	0.76	1.60	0.01148	↓	
UMP	Neg	1	323.0282	32	8	0.77	1.08	0.19633	0.83	1.64	0.19899	↓	

The ionization mode, level of identification (ID), mass to charge ratio (m/z), retention time (RT/s), coefficient of variation in quality control samples [CV(QC)], fold change, VIP score and p value for the two datasets are listed. Arrows indicate the direction of regulation when comparing plasma collected after the RIC intervention to plasma collected before

^a^Pantothenate and propionylcarnitine have been included due to biochemical relevance even though they are slightly less regulated than the inclusion criteria

^b^The corresponding feature was removed in the optimized OPLS-DA model due to a lack of importance

^c^Statistics in validation data based on single analysis of each sample

^d^Alternatively another less common C11 acylcarnitine isomer

^e^Positional isomer not known

^f^Isomer not known

### Quantification of AHB and BHB

A quantitative analysis of AHB was performed based on a method developed to quantify levels of the structurally similar metabolite β-hydroxybutyrate (BHB) (Sørensen et al. 2013). In order to optimize the quantification of AHB, minor changes in the analysis were, however, necessary. Briefly, analysis was performed using a Waters Acquity UPLC coupled to a Waters Xevo TQ-S mass spectrometer both from Waters Corporation (Milford, MA, USA). Separation used a Waters ACQUITY UPLC HSS T3 column (2.1 × 100 mm, 1.7 μm) with a column temperature of 45 °C and an injection volume of 7.5 µL. Mobile phases consisted of water, 0.2% formic acid (A1) and acetonitril, 0.2% formic acid (B1), and a constant flowrate of 0.4 mL/min was used. A gradient elution spanned a total of 5 min being isocratic at 55% B1 during 0.0–3.0 min, linear from 55 to 90% B1 at 3.0–3.1 min, isocratic at 90% B1 during 3.1–3.5 min, linear from 90 to 55% B1 at 3.5–4.0 min, and isocratic at 55% B1 during 4.0–5.0 min. For acquisition, electrospray ionization in negative ion mode was used with a capillary voltage of 2.5 kV. The cone and desolvation nitrogen gas flow were 150 and 600 L/h, respectively, with source temperature of 150 °C and a desolvation temperature of 800 °C. The MRM mode was used for monitoring the transitions BHB (m/z 102.8 > 58.9), AHB (m/z 103.0 > 57.0), and internal standard BHB-^13^C_4_ (m/z 106.8 > 60.9) using cone voltage (V) = 18 and collision energy = 10 (eV) for all transitions.

Samples from the validation set were worked-up using protein precipitation followed by an SPE clean-up procedure in accordance with the published method. The final eluate from SPE was re-dissolved in acetonitrile/0.05% acetic acid (50:50) after evaporation under N_2_. External calibration was performed by spiking of 500, 200, 100, 50, and 5 µM BHB and AHB to a series of plasma samples that underwent the same extraction procedure as the remaining samples. Similarly, QC samples were prepared by spiking BHB and AHB to a plasma sample (100 µM). Stable isotope labeled BHB (BHB-^13^C_4_) was used as internal standard for both BHB and AHB analysis. Calibration curves were created from (1/x) weighted linear regression analysis of internal-standard normalized responses (peak area analyte/peak area internal standard). The concentrations of BHB and AHB were determined by inverse prediction from the sample response value using the regression parameters derived from the linear calibration model with Y-intercept set to zero to account for the endogenous (pre-spike) levels of BHB and AHB in plasma. Analysis performance was evaluated from the retrieval of AHB and BHB in QC samples in accordance with the published method.

### Cell studies of cardioprotection

A HL-1 cardiomyocyte based model was developed for investigation of cardioprotection of either the collected plasma/serum samples or the cell permeable precursor of AHB, methyl α-hydroxybutyrate (MHB). HL-1 cells were cultured in accordance with the provided guidelines from professor Claycomb using T75 culturing flasks supplemented with Claycomb Medium, containing fetal bovine serum (10%), norepinephrine (0.1 mM), l-glutamine (2 mM), and penicillin/streptomycin (100 U/mL:100 µg/mL). Before addition of cells, cultivations flasks were coated with fibronectin (5 µg/mL) dissolved in a gelatin solution (0.02%). Cultivation conditions were 37 °C, 5% CO_2_, and 95% humidity. For experiments, cells of passage 60–63 were seeded into six-well plates ( ∼1 × 10^6^ cells in each well) using the same fibronectin/gelatin coating and allowed to reach 100% confluency and a contractive ability over 2 days. Cells were exposed to 40 min or 1 h of stabilization, followed by 5 h 30 min of ischemia and 2 h of reperfusion in accordance with previous optimization studies (data not shown). For stabilization and reperfusion, cells were incubated at normal atmosphere in the presence of stabilization buffer solution (CaCl_2_ dihydrate 1.25 mM, MgCl hexahydrate 1 mM, Hepes 6 mM, NaH_2_PO_4_ dihydrate 0.9 mM, NaCl 137 mM, KCl 6 mM, glucose 10 mM, pH = 7.4) (2 mL during stabilization and 2 mL for reperfusion). Simulated ischemia was inflicted by placing the six-well plates without lid in a hypoxic chamber containing a N_2_ atmosphere. Furthermore, cells were exposed to an ischemia buffer solution (CaCl_2_ dihydrate 1.25 mM, MgCl hexahydrate 1 mM, Hepes 6 mM, NaH_2_PO_4_ dihydrate 0.9 mM, NaCl 120 mM, KCl 8 mM, Na-Lactate 20 mM, pH = 6.8) (2 mL). The oxygen level of the ischemia buffer was <15% compared to normoxia levels within 20 min after placement in the hypoxic chamber (data not shown). During all three phases cells were incubated in the presence of either plasma/serum (1%) or MHB (0.1, 0.5, 1, 5 mM) (Fig. 1b). Cellular damage was, subsequently, measured as a propidium iodide/hoechst33342 (PI/HOE) ratio at 530/620 nm (PI) and 350/460 nm (Hoechst) using a PHERAstar FS plate readerfrom BMG Labtech (Ortenberg, Germany).

Using the untargeted metabolomics method described earlier, the intracellular content of AHB, BHB, and MHB was measured in cells treated with MHB during stabilization, ischemia and reperfusion. Cells were harvested after reperfusion following an in-house protocol. Shortly, buffer solution was removed and the adherent cells were quickly washed with water before lifting them using a cell scraper in ice-cold methanol/water (80:20) (1 mL). Cell solution was transferred to tubes and wells were washed with ice-cold methanol/water (80:20) (1 mL) to improve collection of cells. To ensure cell membrane breakage, collected cells were shaken (1000 Hz, 5 min) before centrifugation (1000*g*, 5 min, 4 °C) and transfer of the supernatant to a second vial kept on ice. The extraction was repeated twice and extracts were pooled before freezing (−80 °C) until analysis. Prior to analysis, extracts were evaporated under N_2_ to dryness and re-suspended in 0.1% formic acid (100 µL).

## Results and discussion

### Cardiomyocyte protection by RIC plasma or serum

Prior to the metabolomics study, a cell based model was used to examine the bioactivity of plasma or serum collected after the RIC stimulus. For this purpose, contracting murine HL-1 cardiomyocytes, previously shown capable of obtaining a conditioned phenotype, were used (Claycomb et al. 1998; Elshenawy et al. 2013; Facundo et al. 2006; Naydenova et al. 2008; White et al. 2004). During the entire simulated ischemia and reperfusion protocol, the cells were exposed to the human plasma or serum collected either before (control) or after (conditioned) RIC (Fig. 1b). Using this cell model, conditioned plasma induced a minor reduction in cell death, as quantified by the PI/HOE ratio, compared to control plasma. This reduction was, however, statistically non-significant (results not shown). A similar observation has been made by others, who however also noticed that ischemic conditioned serum leads to more clear results compared to plasma (Weber et al. 2015; Zitta et al. 2012). Acknowledging this, the assay was repeated using serum collected before and after RIC from four randomly selected volunteers. This led to a statistically significant reduction of the PI/HOE ratio (fold = 0.79, p = 0.0048, n = 4) in cells exposed to conditioned serum, validating the bioactivity hereof. The model showed less cardioprotective capacity than our previous studies of human to rabbit transfer in the isolated rabbit heart ex vivo Langendorff model using plasma dialysate (Jensen et al. 2012; Michelsen et al. 2012). This might be due to unfavorable cross-species interactions between proteins present in the plasma/ serum which are absent in the dialysate, though it can also be model dependent.

### Elucidation, identification and validation of metabolites regulated in conditioned plasma

As we demonstrated a statistically significant reduction in cell death using RIC serum, we proceeded to characterize the influence of RIC on the plasma metabolome using untargeted LC-MS based metabolomics. Plasma was preferred in favor of serum because a faster work-up was achievable which potentially could minimize changes to the metabolome during the procedure.

Using the calibration set, a total of 3360 (79%) detected features met the criterion of a CV(QC) < 30% in the positive ionization mode. In the negative ionization mode 2411 (80%) detected features fulfilled this criterion. Initial principal component analysis models of data from the calibration study, analyzed both in positive and negative ionization mode, failed to separate control and conditioned plasma samples (Fig. S2). The QC samples clustered nicely in the middle of the plots, indicating that the technical variation was smaller than biological variability. Subsequently, a supervised OPLS-DA model was built with the model parameters R^2^X(cum) = 0.409, R^2^Y (cum) = 0.997, Q^2^(cum) = 0.809, for positive ionization mode covering 11.8% of total variation and R^2^X(cum) = 0.305, R^2^Y(cum) = 0.989, Q^2^(cum) = 0.753 for negative ionization mode explaining 13.7% of total variation (R^2^ investigates the goodness of fitting while Q^2^ investigates goodness of prediction). This indicated only a modest change in the plasma metabolites following RIC. Based on this model, VIP values were calculated, and using the earlier mentioned selection criteria, features were selected for further investigation. The selected features were finally validated by examining that they had a similar upregulation or downregulation in both calibration and validation data sets. Out of the initially selected features 31 were finally found to be regulated in the same direction, confirming their importance in both sets, while the remaining were discarded as irrelevant regulations to the study of RIC. Of the 31 features identification of 21 was possible to level 1–3 according to guidelines of the Metabolomics Standard Initiative (Table 1) (Sumner et al. 2007). Ten features/metabolites remained unknown (Table S1).

Overall, the selected features possessed moderate fold changes (0.67–1.23) and only few were statistically significantly altered, demonstrating a mild impact of RIC on the plasma metabolome. This was also indicated by the lack of separation of groups in the PCA model. A majority of the selected metabolites were downregulated including adenosine, hypoxanthine, UMP, and medium to long-chain acyl carnitines. Among the upregulated metabolites, α-hydroxybutyrate (AHB), 2-hydroxy-3-methylbutyrate, pantothenate and succinyladenosine were identified. In addition, the regulation of AHB was the most pronounced alternation revealed in the plasma samples, showing a fold change of 1.23, VIP score of 1.41, and a p value <0.00001 for samples included in the calibration set.

**Fig. 2:**
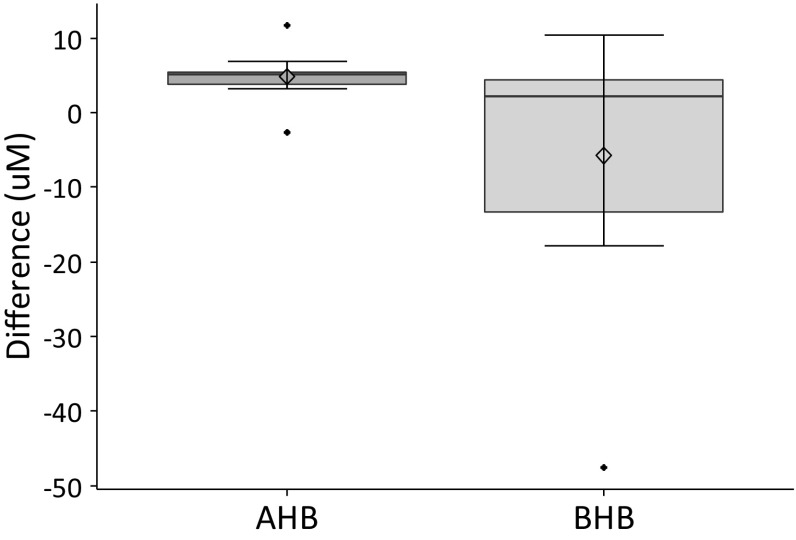
Differences (µM) in quantified levels of α-hydroxybutyrate (AHB) and β-hydroxybutyrate (BHB) in plasma samples collected before and after RIC, calculated in a pairwise fashion

Subsequent, LC-MS/MS based quantification of AHB and the structurally similar metabolite β-hydroxybutyrate (BHB), confirmed a significant upregulation of AHB (fold = 1.18, p = 0.0021) in plasma samples of the validation set, while BHB remained unchanged after the RIC stimulus (fold = 0.96, p = 0.3224) (Fig. 2).

### Biological interpretation

As RIC is known to have a circulating as well as a neuronal component, it is possible that some of the findings reflect metabolic changes remote from the arm undergoing ischemic conditioning. The likelihood of observing this is further raised by the sampling strategy where blood samples, collected before and after the RIC stimulus, were drawn from the contralateral arm remote from the ischemic tissue. However, this strategy at the same time ensures that possible observations more likely reflect true circulating levels of metabolites and not hypothetically more prominent local changes if samples had been withdrawn from the arm undergoing RIC maneuver. Obviously, this duality can complicate the interpretation of the changes observed in the plasma metabolome and at the same time pose challenges to the analysis as the changes, as observed, likely will be rather subtle (Murry et al. 1990).

#### Adenosine, hypoxanthine, UMP and succinyladenosine

Since the discovery that adenosine and adenosine signaling, via its cellular receptors, can mediate cardioprotection, focus has targeted the role of this prominent metabolite in RIC (Chaudary et al. 2004; Lim and Hausenloy 2012; Sivaraman et al. 2015; Surendra et al. 2013). While an increased cellular uptake of adenosine following RIC has been shown (Chaudary et al. 2004) and might explain the downregulation of the metabolite in conditioned plasma, this contradicts the notion that adenosine is released to the blood stream to meditate protection as well as studies measuring elevated adenosine levels in effluent after RIC (Chaudary et al. 2004; Surendra et al. 2013). During the present study, downregulation of adenosine in plasma was found using both positive and negative ionization mode and this was mirrored by a similar regulation of the nucleotide metabolites hypoxanthine and UMP. Albeit statistically insignificant, both compounds remained regulated in a similar direction in both the calibration and validation set. Finally, we demonstrated that succinyladenosine was significantly upregulated in the calibration samples. Despite the subtle changes, our general interpretation is that levels of adenosine and metabolites reflect tissue ischemia and an impaired oxidative metabolism in the arm undergoing ischemia–reperfusion maneuver.

#### Acylcarnitines (medium and long chain)

The most prominent group of downregulated metabolites after RIC were the medium to long chained acylcarnitines (C10-14). All of these compounds were consistently downregulated approximately 20% following conditioning, showing a similar influence on the OPLS-DA model (VIP ≈ 1) and significance levels (p ≈ 0.03–0.04).

A recent study demonstrated that plasma levels of acylcarnitines reflect intracellular levels of these compounds during fasting (Makrecka et al. 2014) and may suggest a similar correlation in the present study. Whether this reflects an organismal downregulation or a more prominent localized effect in the arm undergoing RIC maneuver is unknown. Being responsible for the transportation of fatty acids into mitochondria for β-oxidation, acylcarnitines play an important role in the energy generation in the heart and other organs (Makrecka et al. 2014). During ischemia, long-chain acylcarnitines accumulate in the ischemic tissue, though ischemic conditioning has been proposed to prevent such an accumulation (Ford et al. 1996; Simkhovich et al. 1993). The downregulation of the acylcarnitines might reflect this phenomenon. It has also been proposed that fatty acid dependent respiration is inhibited following ischemic conditioning possibly also leading to a lower plasma level of acylcarnitines (Nadtochiy et al. 2015).

#### Pantothenate (vitamin B_5_)

Vitamin B_5_ was one of the few metabolites significantly upregulated (p = 0.02044). This is in accordance with a targeted metabolomics study of mice hearts mounted in an ex vivo isolated mouse heart Langendorff setup, showing a two-fold upregulation after ischemic conditioning (Nadtochiy et al. 2015). The regulation of pantothenate during ischemia, i.e. RIC maneuver, might be influenced by mitochondrial levels of acylcarnitines and coenzyme A as these metabolites are known to regulate activity of pantothenate kinase (Leonardi et al. 2007; Rock et al. 2003).

#### Sphinganine 1-phosphate and sphingosine 1-phosphate

Sphingosine 1-phosphate is an endogenous cardioprotective agent that is released from cardiomyocytes during ischemic preconditioning and postconditioning (Jin et al. 2004, 2008; Keul et al. 2016; Vessey et al. 2009). Our data indicate that both sphinganine 1-phosphate and sphingosine 1-phosphate influence the separation of the RIC samples in the OPLS-DA analysis (VIP = 1.06 and 1.10, respectively), although the difference between IPC and control was statistically insignificant (p = 0.28048 and 0.22922, respectively). Since both compounds were found in the analysis and since the data have been verified in two consecutive studies, they could, however, still be of relevance. Our findings indicate that both compounds are slightly downregulated following RIC and therefore questions whether sphingosine 1-phosphate or sphinganine 1-phosphate are circulating molecules mediating RIC.

#### α-Hydroxybutyrate (AHB), 2-hydroxy-3-methylbutyrate and propionylcarnitine

**Fig. 3:**
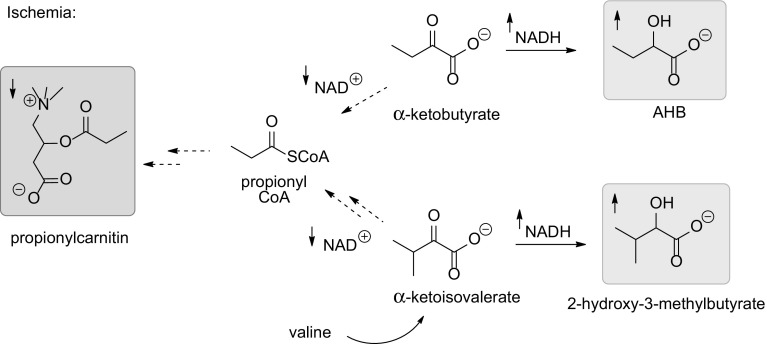
Metabolism of α-keto acids during ischemia: Increased formation of NADH and reduced α-keto acids AHB and 2-hydroxy-3-methylbutyrate and concomitantly downregulated NAD^+^ and propionyl coenzyme A derived propionylcarnitin during ischemia

Of the plasma metabolites, AHB increased most significantly (p = 0.00001) in the range of 20–25%. The significance level exceeded the other regulated metabolites with orders of magnitude. The increase was verified by independent quantitative analysis. AHB is the NADH mediated reduction product of α-ketobutyrate. During normoxic conditions, AHB is oxidatively decarboxylated by an NAD^+^ dependent alpha-keto acid dehydrogenase complex to yield propionyl CoA (Lapointe and Olson 1985). As the NADH/NAD^+^ ratio is increased during hypoxia, due to impaired oxidative phosphorylation, α-ketobutyrate is likely diverted into AHB rather than into propionyl CoA (Fig. 3). This is in accordance with the decreased levels of propionylcarnitine that we have detected in the current study; given acylcarnitine levels reflect acyl CoA levels. Similarly, 2-hydroxy-3-methylbutyrate is the NADH mediated reduction product of α-ketoisovalerate, a catabolic metabolite of valine (Fig. 3) (Walker et al. 1996). Under normoxic conditions valine is ultimately also degraded to propionyl CoA, i.e. hypoxia could also lead to lower levels of this as well as the coupled carnitine metabolite as mentioned above.

AHB concentrations have previously been suggested as an early marker for insulin resistance through an increased β-oxidation and an elevated NADH/NAD^+^ ratio (Gall et al. 2010). Similarly, accumulation of AHB in blood and urine and an increase in NADH/NAD^+^ ratio in the cytoplasm has previously been related to hypoxic conditions such as extreme exercise (Landaas and Pettersen 1975; Malagrino et al. 2016; Pechlivanis et al. 2010, 2014). In our study, plasma NADH was below limit of detection (LOD), while NAD^+^ was detected at a very low intensity (<5000 counts) causing a large measurement variance (CV(QC) = 29%). Even so, downregulation of NAD^+^ (fold = 1.62, VIP = 1.59, p = 0.03467) was demonstrated in the calibration samples, indicating a possible elevated NADH/NAD^+^ ratio and thereby a mechanism that could increase AHB formation. As we did not detect a regulation of lactate following RIC, as is usually the case after e.g. intense exercise, it appears as AHB is a more sensitive plasma biomarker of transient and local tissue ischemia than lactate (Pechlivanis et al. 2014). The reason is likely the orders of magnitude higher levels of plasma lactate (mM) compared to AHB (uM), resulting in a relatively higher perturbation of AHB than lactate levels during hypoxia.

It can also be noted that the structurally similar liver metabolite BHB, that is the direct NADH derived reduction product of acetoacetate, did not change following RIC (Fig. 2), supporting that the elevated plasma levels of NADH and AHB originate from the arm undergoing ischemic conditioning.

### Cell based investigation of AHB

**Fig. 4:**
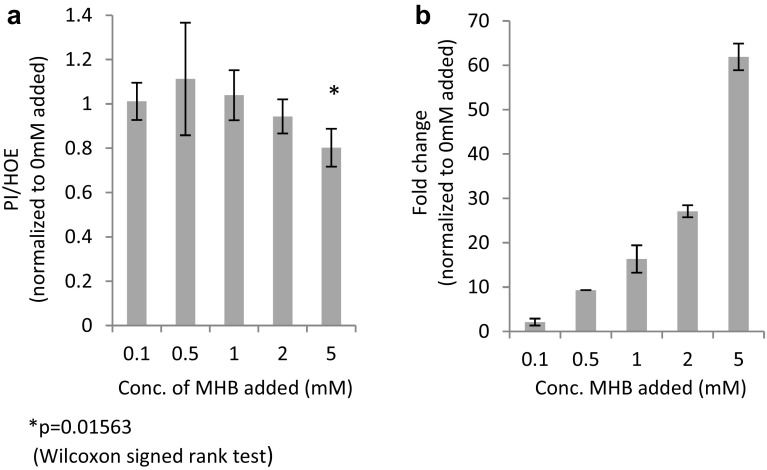
Cellular damage (n = 7) (**a**) measured as propidium iodide/hoechst33342 (PI/HOE) after reperfusion in the presence of 0.1, 0.5, 1, 2, and 5 mM (R)/(S)-methyl-2-hydroxybutanoate (MHB) during stabilization, simulated ischemia and reperfusion of cells and fold change (**b**) of intercellular concentration of α-hydroxybutyrate (AHB) (n = 2) measured semi-quantitatively in harvested cells (significance not calculated due to low number of included samples)

Owing to the significance of the upregulation of AHB following the relatively mild ischemic exposure, it seemed reasonable to assume that this metabolite plays a physiological role during ischemia–reperfusion. This hypothesis was further supported by the fact that the structural isomer, BHB, is a known cardioprotective metabolite (Zou et al. 2002). To investigate this, HL-1 cardiomyocytes were grown in a media supplemented with increasing concentrations of the cell permeable AHB precursor, methyl α-hydroxybutyrate (MHB), during a prolonged ischemia and reperfusion challenge. A dose–dependent and significant reduction of the PI/HOE ratio by up to 20% showed diminished cellular damage of MHB treated cells (p = 0.01563, fold = 0.80, n = 7) (Fig. 4a) and suggested that AHB indeed could be a novel cardioprotective metabolite. To support that the effect was related to AHB and not the methyl ester, cells treated with increasing concentrations of MHB were harvested after reperfusion, and the intracellular levels of AHB, BHB, and MHB was analysed using LC-qTOFMS. The results revealed a dose-dependent increase in intracellular concentrations of AHB while BHB and MHB was undetectable (Fig. 4b). As the levels of MHB ameliorating cardiomyocyte death exceeded plasma levels of AHB (1–5 mM vs 50 µM) significantly, the results indicate that AHB is not a circulating mediator of cardioprotection, but more likely can be part of a cell inherent system to protect against ischemia–reperfusion damages. Hence, the low but significant increase of plasma AHB might reflect a local and more pronounced cellular accumulation of AHB in the arm undergoing the RIC maneuver. Future studies are currently underway to clarify this as well as the mechanism of protection.

## Conclusion

The present study shows that untargeted metabolomics can be used to investigate the influence of RIC on the plasma metabolome. This was possible even though the changes of the biological system, and hence the metabolome, were rather subtle and only few metabolites were significantly altered. Limitations of the study does, however, include the minor cohorts and statistics could be improved using larger sample sets. Despite hereof, findings were rediscovered in a validation set, and the results collectively seem to present a coherent picture of the biological changes occurring in the arm undergoing ischemic conditioning. Moreover, one regulated metabolite, AHB, was found to possess biological activity of relevance to ischemic/reperfusion damage supporting the validity of the findings.

Our results indicate that neither adenosine nor sphingosine-1-phosphate are circulating mediators of cardioprotection following RIC. Furthermore, they show a universal downregulation of medium-chain and long-chain acyl carnitines which might reflect a well-known mitochondrial uptake of carnitines during ischemia. Most importantly however, AHB was identified as the most significantly regulated metabolite, likely reflecting that arm ischemia induces accumulation of NADH followed by α-ketobutyrate reduction to AHB. The sensitivity of AHB as an ischemia marker seems higher than that of lactate, as the latter was uninfluenced by the RIC maneuver during this study. Finally, it was found that AHB could be a novel cardioprotective metabolite capable of reducing ischemia/reperfusion injuries. This latter finding warrants further attention to both clarify a possible physiological role of AHB in ischemic tissue, as well as to examine AHB and its esters as possible novel agents to ameliorate ischemia/reperfusion damage in ex vivo models and in vivo. These studies are currently underway in our laboratories.

## Electronic supplementary material

Below is the link to the electronic supplementary material.

Supplementary material 1 (DOCX 72 KB)
