# Supplementary material for: Untargeted metabolomics reveals a mild impact of remote ischemic conditioning on the plasma metabolome and α-hydroxybutyrate as a possible cardioprotective factor and biomarker of tissue ischemia
Source: Metabolomics. 2017 Apr 17;13(6):67. doi: 10.1007/s11306-017-1202-2 (PMC5392534; doi:10.1007/s11306-017-1202-2)
Supplement: Supplementary file 1 — Supplementary material 1 (DOCX 72 KB) [file 11306_2017_1202_MOESM1_ESM.docx]

Untargeted Metabolomics Reveals a Mild Impact of Remote Ischemic Conditioning on the Plasma Metabolome and α-Hydroxybutyrate as a Possible Cardioprotective Factor and Biomarker of Tissue Ischemia

Mia Roest Laursen^†^, Jakob Hansen^†^, Casper Elkjær^‡^, Ninna Stavnager^†^, Camilla Bak Nielsen^†^, Kasper Pryds^‡^, Jacob Johnsen^‡^, Jan Møller Nielsen^‡^, Hans Erik Bøtker^‡,*^ and Mogens Johannsen^†,*^

^†^Department of Forensic Medicine, Section for Forensic Chemistry, Aarhus University, Aarhus N, Denmark.

^‡^Department of Cardiology, Aarhus University Hospital, Aarhus C, Denmark

**Table of contents:**

**Supporting information Page#**

Figure S1 S2

Figure S2 S2

Table S1 S3

Table S2 S3

Table S3 S4

**Figure S1** Representative chromatograms for analyses in a) positive ionization mode and b) negative ionization mode.

**a)**

**b)**

**Figure S2** PCA score plot for a) positive ionization mode and b) negative ionization mode, containing all detected features with CV(QC)<30. A nice clustering of QC samples (gray) validates the system performance throughout analyses, while no separation of samples collected before (red) and after (blue) was seen.

**a)** **b)**

**Table S1** Evaluation of instrument stability for positive ionization mode. The predefined requirements (m/z deviation<10 ppm, rt deviation<5 sec and peak area CV<0.30) were met for all metabolites.

|  | Instrument control samples (n=2) | | |  | Quality control samples (n=10) | | |
| --- | --- | --- | --- | --- | --- | --- | --- |
|  | m/z deviation (ppm) | rt deviation  (s) | Peak area  (CV) |  | m/z deviation (ppm) | rt deviation  (s) | Peak area  (CV) |
| Butanoyl carnitine | 6.49 | 0.50 | 0.125 |  | 1.43 | 0.314 | 0.066 |
| Citric acid | 5.83 | 0.00 | 0.047 |  | 1.48 | 0.464 | 0.099 |
| Glutamaic acid | 6.43 | 0.33 | 0.121 |  | 2.30 | 1.012 | 0.031 |
| Inosine | 6.62 | 0.25 | 0.112 |  | 1.61 | 0.747 | 0.034 |
| Palmitoyl carnitine | 5.19 | 4.72 | 0.145 |  | 2.75 | 3.504 | 0.138 |
| Valine | 4.63 | 0.25 | 0.087 |  | 2.06 | 0.402 | 0.031 |
| Xanthine | 7.34 | 0.42 | 0.114 |  | 2.73 | 0.333 | 0.097 |

**Table S2** Evaluation of instrument stability for negative ionization mode. The predefined requirements (m/z deviation<10 ppm, rt deviation<5 sec and peak area CV<0.30) were met for all metabolites.

|  | Instrument control samples (n=2) | | |  | Quality control samples (n=7) | | |
| --- | --- | --- | --- | --- | --- | --- | --- |
|  | m/z deviation (ppm) | rt deviation  (s) | Peak area  (CV) |  | m/z deviation (ppm) | rt deviation  (s) | Peak area  (CV) |
| Adenosine | 0.04 | 0.29 | 0.032 |  | 1.47 | 0.89 | 0.027 |
| Citric acid | 0.00 | 0.00 | 0.123 |  | 0.94 | 0.39 | 0.139 |
| Glutamic acid | 0.02 | 0.35 | 0.020 |  | 1.27 | 0.66 | 0.179 |
| Hippuric acid | 0.02 | 0.00 | 0.013 |  | 1.53 | 1.04 | 0.032 |
| Histidine | 0.01 | 0.19 | 0.038 |  | 4.91 | 2.32 | 0.193 |
| Inosine | 0.02 | 0.00 | 0.045 |  | 0.88 | 2.58 | 0.034 |
| Malic acid | 0.01 | 0.88 | 0.009 |  | 2.66 | 0.56 | 0.118 |
| Succinic acid | 0.02 | 0.08 | 0.025 |  | 4.36 | 0.68 | 0.058 |
| Tryptophane | 0.11 | 0.25 | 0.029 |  | 0.91 | 0.25 | 0.047 |
| Xanthine | 0.02 | 0.02 | 0.028 |  | 2.66 | 0.61 | 0.070 |

**Table S3** Unidentified metabolites

|  |  |  |  |  |  |  |  |  |  |  |  | Calibration set | | | | |  | Validation set | | | | |  |  |
| --- | --- | --- | --- | --- | --- | --- | --- | --- | --- | --- | --- | --- | --- | --- | --- | --- | --- | --- | --- | --- | --- | --- | --- | --- |
| Metabolite |  | Ion.Mode |  | ID.level |  | m/z |  | rt |  | CV(QC) |  | Fold |  | VIP |  | p |  | Fold |  | VIP |  | p |  | Regulation |
| Unknown |  | Pos |  | 4 |  | 173,0782 |  | 180 |  | 9 |  | 0,90 |  | 1,22 |  | 0,02242 |  | 0,97 |  | 0,83 |  | 0,50877 |  | ↓ |
| Unknown |  | Pos |  | 4 |  | 185,1283 |  | 210 |  | 4 |  | 1,10 |  | n.a.^a^ |  | 0,00157 |  | 1,05 |  | 0,23 |  | 0,02888 |  | ↑ |
| Unknown |  | Pos |  | 4 |  | 286,2016 |  | 317 |  | 3 |  | 1,12 |  | n.a.^a^ |  | 0,03957 |  | 1,04 |  | 0,02 |  | 0,82572 |  | ↑ |
| Unknown |  | Pos |  | 4 |  | 359,0462 |  | 44 |  | 5 |  | 1,11 |  | 1,03 |  | 0,00658 |  | 1,02 |  | 0,43 |  | 0,22182 |  | ↑ |
| Unknown |  | Pos |  | 4 |  | 153,0657 |  | 124 |  | 6 |  | 1,13 |  | 0,75 |  | 0,01289 |  | 1,06 |  | 0,64 |  | 0,05755 |  | ↑ |
| Unknown |  | Pos |  | 4 |  | 239,0914 |  | 291 |  | 3 |  | 1,12 |  | n.a.^a^ |  | 0,02510 |  | 1,03 |  | 0,12 |  | 0,27217 |  | ↑ |
| Unknown |  | Pos |  | 4 |  | 527,1587 |  | 28 |  | 4 |  | 0,67 |  | 1,60 |  | 0,08679 |  | 0,95 |  | 0,88 |  | 0,36107 |  | ↓ |
| Unknown |  | Neg |  | 4 |  | 281,1005 |  | 241 |  | 4 |  | 1,11 |  | 0,89 |  | 0,01558 |  | 1,09 |  | 0,70 |  | 0,18115 |  | ↑ |
| Unknown |  | Neg |  | 4 |  | 541,2653 |  | 327 |  | 9 |  | 1,22 |  | n.a.^a^ |  | 0,00743 |  | 1,12 |  | 0,93 |  | 0,12801 |  | ↑ |
| Unknown |  | Neg |  | 4 |  | 539,2496 |  | 335 |  | 8 |  | 1,17 |  | 0,81 |  | 0,01829 |  | 1,12 |  | 0,49 |  | 0,32317 |  | ↑ |

^a^The corresponding feature was removed in the optimized OPLS-DA model due to a lack of importance
